# Long Non-coding RNA *MEG3* Activated by Vitamin D Suppresses Glycolysis in Colorectal Cancer *via* Promoting c-Myc Degradation

**DOI:** 10.3389/fonc.2020.00274

**Published:** 2020-03-11

**Authors:** Siyu Zuo, Lei Wu, Yi Wang, Xiaoqin Yuan

**Affiliations:** ^1^Department of Anatomy, Histology, and Embryology, Nanjing Medical University, Nanjing, China; ^2^Department of Clinical Medicine, First Clinical Medicine College, Nanjing Medical University, Nanjing, China; ^3^Key Laboratory for Aging and Disease, Nanjing Medical University, Nanjing, China

**Keywords:** colorectal cancer, maternally expressed gene 3 (MEG3), glycolysis, c-Myc, vitamin D

## Abstract

Colorectal cancer (CRC), a common tumor, is characterized by a high mortality rate. Long non-coding RNA *maternally expressed gene 3 (MEG3)* serves a regulatory role in the carcinogenesis and progression of several types of cancer; however, its role in CRC remains largely unknown. The aim of this study was to explore the regulatory role and mechanism(s) of *MEG3* in CRC. The Warburg effect or aerobic glycolysis is characteristic of the metabolism of tumor cells. To determine the effect of *MEG3* on glycolysis of CRC cells, we used an XF analyzer to perform glycolysis stress test assays and found that overexpression of MEG3 significantly inhibited glycolysis, glycolytic capacity, as well as lactate production in CRC cells, whereas knockdown of MEG3 produced the opposite effect. Mechanistically, overexpression of MEG3 induced ubiquitin-dependent degradation of c-Myc and inhibited c-Myc target genes involved in the glycolysis pathway such as lactate dehydrogenase A, pyruvate kinase muscle 2, and hexokinase 2. Moreover, we found that *MEG3* can be activated by vitamin D and vitamin D receptor (VDR). Clinical data demonstrated that *MEG3* was positively associated with serum vitamin D concentrations in patients with CRC. We found that 1,25(OH)_2_D_3_ treatment increased *MEG3* expression, and knockdown of VDR abolished the effect of MEG3 on glycolysis. These results indicate that vitamin D-activated *MEG3* suppresses aerobic glycolysis in CRC cells *via* degradation of c-Myc. Thus, vitamin D may have therapeutic value in the treatment of CRC.

## Introduction

Colorectal cancer (CRC), the third most commonly diagnosed cancer, has a high cancer-related mortality rate worldwide (1, 2). In China, CRC is the fourth most common cancer, with 376,000 new patients diagnosed in 2015, leading to about 191,000 deaths (3). However, the molecular mechanisms underlying CRC progression are not fully understood. More research is needed to discover and develop effective biomarkers and targets for diagnosis and treatment of CRC. Recently, accumulating evidence has shown that long non-coding RNAs (lncRNAs), non-coding RNA transcripts longer than 200 nucleotides (4), are expressed differentially in various cancers including CRCs, suggesting that lncRNAs have roles in tumorigenesis and tumor metastasis (5–7). Among them, *maternally expressed gene 3* (*MEG3*) has been reported to be aberrantly expressed in CRC and may act as a tumor suppressor (8–10). However, the mechanism of action of *MEG3* in CRC requires further investigation.

It has been demonstrated that most cancer cells have altered energy metabolism characterized by glycolysis with lactate production and a higher uptake of glucose as the main source of energy even in the presence of oxygen, well-known as the “Warburg effect” (11, 12). Under normoxic conditions, glycolysis is commonly driven by c-Myc (13, 14), which upregulates glycolytic enzymes such as lactate dehydrogenase A (LDHA) and hexokinase 2 (HK2) (15–17). Many non-coding RNAs have been reported to be involved in the regulation of cancer metabolism (18). For example, lncRNA *PVT1* promotes glycolysis and tumor progression by regulating the miR-497/HK2 axis in osteosarcoma (19), whereas lncRNA-*MIF* inhibits aerobic glycolysis and tumorigenesis by suppressing c-Myc and miR-586 in cancer cells (20).

In this study, we demonstrate that *MEG3* activated by vitamin D and vitamin D receptor (VDR) suppresses activation of glycolysis by promoting c-Myc degradation under normoxic conditions.

## Materials and Methods

### Clinical Samples

A total of 80 CRC tissue samples and corresponding adjacent normal mucosal samples were collected at the First Affiliated Hospital of Nanjing Medical University. All samples were snap-frozen in liquid nitrogen immediately after collection and stored at −80°C until total RNA was extracted. The clinicopathological characteristics of the patients with CRC from whom the samples were obtained are summarized in Table 1. This project was approved by the Research Ethics Committee of Nanjing Medical University [Approval ID: (2016)640].

**Table 1:** Association of *maternally expressed gene 3* (*MEG3*) expression with clinicopathological variables in patients with colorectal cancer (CRC) (*n* = 80).

**Feature**	**Total**	**Expression of MEG3**		***P***
		**Low**	**High**	
**Gender**				
Female	35	25	10	1
Male	45	33	12	
**Age (years)**				
≤60	39	31	8	0.214
>60	41	27	14	
**Clinical grade**				
I and II	42	25	17	0.011
III and IV	38	33	5	
**T**				
1 and 2	19	14	5	1
3 and 4	61	44	17	
**N**				
0	41	24	17	0.016
1	19	17	2	
2	20	17	3	
M				
**No**	76	54	22	0.571
Yes	4	4	0	
**Tumor size (cm)**				
≤5	59	43	16	1
>5	21	15	6	

### Cell Culture

The human CRC cell lines DLD-1 and RKO were purchased from the Shanghai Institute of Cell Biology, Chinese Academy of Sciences (Shanghai, China), and were maintained in RPMI-1640 (HyClone, Logan, UT, USA), supplemented with 10% fetal bovine serum (FBS; Gibco, Grand Island, NY, USA), 100 U/ml penicillin, and 0.1 mg/ml streptomycin (Gibco). All cell lines were cultured in a humidified incubator of 5% CO_2_ at 37°C. Cells were collected for further studies after treatment with cycloheximide (CHX) and MG132 (MedchemExpress, Monmouth Junction, NJ, USA) for various periods of time.

### RNA Extraction and Real-Time Polymerase Chain Reaction

Total RNA was extracted from frozen tissues with TRIzol reagent (Invitrogen, Grand Island, NY, USA) and reverse transcribed to complementary DNA (cDNA) by using a PrimeScript™ 1st Strand cDNA Synthesis Kit (Takara Bio, Shiga, Japan) according to the user's manual. Real-time polymerase chain reaction (RT-PCR) was performed with gene-specific primers to determine the relative expression of genes of interest using SYBR green reagents (Takara Bio) in an ABI 7300 sequence detector (Applied Biosystems, Foster City, CA, USA). Glyceraldehyde 3-phosphate dehydrogenase (GAPDH) or ACTIN mRNA was used for normalization. The PCR primers used in this study are listed in Table S1.

### Small Interfering RNA and Plasmid Transfection

The cDNAs encoding *MEG3* and *VDR* were synthesized and cloned into the pcDNA3.1 vector to construct pcDNA-MEG3 and pcDNA-VDR vector, respectively. Empty pcDNA3.1 vector was used as the control. The small interfering RNA (siRNA) targeting *MEG3* (siRNA-MEG3) and negative control siRNA (siRNA-NC) were synthesized by RiboBio (Guangzhou, China). The siRNA sequences for *MEG3* were as follows: *MEG3* siRNA1: sense: 5′-GGAUGGCACUUGACCUAGA-3′, antisense: 5′-UCUAGGUCAAGUGCCAUCC-3′; siRNA2: sense: 5′-GAACCAUUCUGUUAUUCUU-3′, antisense: 5′-AAGAAUAACAGAAUGGUUC-3′; and siRNA3: sense: 5′-GGUUAAGUCUCUUGAAAGA-3′, antisense: 5′-UCUUUUCAAGAGACUUAACC-3′. The target sequence of *VDR*-specific short hairpin RNA (shRNA) (sh-VDR) was: 5′-TCCAGTTCGTGAATGAT-3′. A non-silencing shRNA (sh-NC) oligonucleotide was used as a negative control. DLD-1 or RKO cells were transfected with plasmid vectors using Lipofectamine 2000 (Invitrogen) and transfected with siRNA using Lipofectamine RNAiMAX (Invitrogen, Waltham, MA, USA), according to the manufacturer's protocol.

### CCK8 Assays

Cell proliferation was detected using a Cell Counting Kit 8 (Donjindo, Kumamoto, Japan). Briefly, cells (3 × 10^3^) transfected with either siRNA or plasmid were seeded into 96-well plates, and cell growth was determined every 24 h for 4 days in accordance with the manufacturer's protocol. The absorbance at a wavelength of 450 nm was measured using a microplate reader (Bio-Tek, Winooski, VT, USA).

### Colony Formation Assays

Transfected cells with siRNA or plasmid were seeded in six-well plates at 600 cells per well and 1,000 cells per well, respectively, and cultured for 10–15 days with replacement with new medium every 3 days. Colonies were fixed, stained with crystal violet, and photographed. For each treatment group, wells were counted in triplicate.

### Transwell Assays

Cell migration and invasion assays were carried out using 24-well Transwell chambers with 8-μm pore size polycarbonate membrane (Corning, NY, USA). A total of 6 × 10^4^ or 8 × 10^4^ cells in 200 μl serum-free medium were seeded in the upper chambers coated without or with Matrigel (BD Biosciences, NY, USA) for migration or invasion assays, respectively, after which 600 μl medium with 10% fetal bovine serum (FBS) (20% FBS for invasion assays) was added to the lower chambers. The cells on the upper surfaces of the membranes were removed 36–48 h later. Cells on the bottom surfaces of the membranes were fixed, stained with 0.1% crystal violet, and counted in five fields using a Zeiss microscope (Melville, NY, USA).

### Western Blot Analysis

Lysates from cells were separated by sodium dodecyl sulfate (SDS)-polyacrylamide gel electrophoresis, transferred onto polyvinylidene fluoride (PVDF) membranes (PerkinElmer, Boston, MA, USA), and blotted with primary antibodies, followed by horseradish peroxidase (HRP)-conjugated secondary antibody. The primary antibodies used were anti-c-Myc and anti-HK2 (Abcam, Cambridge, MA, USA) and anti-PKM2 (Affinity Biosciences, OH, USA); and anti-LDHA, anti-GAPDH, and anti-actin (Santa Cruz, CA, USA); and anti-FBXW7 (ABclonal, Wuhan, China).

### Glycolysis Stress Test

The extracellular acidification rate (ECAR) was measured using a Seahorse XF96 Analyzer Glycolysis (Seahorse Bioscience, Santa Clara, USA) according to the manufacturer's protocol. Briefly, DLD-1 or RKO cells in 10% FBS RPMI-1640 were seeded in XF 96-well plates and incubated at 37°C in a 5% CO_2_ humidified atmosphere overnight. The cells were then incubated in the glycolysis stress test medium without glucose, and the ECAR was measured. Following this, D-glucose (10 mM), oligomycin (1 μM), and 2-deoxyglucose (100 mM) were added into the wells at the indicated time points; meanwhile, corresponding ECARs were assessed. The ECAR values are presented as the mean ± SD of experimental triplicates. The key variables of glycolysis and glycolytic capacity were analyzed using XF Glycolysis Stress Test software.

### Lactate Production Assay

CRC cells transfected with siRNA or plasmid or treated with vitamin D were cultured, and the culture medium was collected 24 h later. Lactate production was quantitated using a lactate assay kit (Jiancheng, Nanjing, China) according to the manufacturer's instructions. Total viable cell numbers were used for normalization.

### Serum Vitamin D Measurement

Serum 25(OH)D_3_ concentrations were measured by chemiluminescence using an ADVIA Centaur Vitamin D Total (VitD) Assay kit (Siemens Healthineers, Erlangen, Germany) according to the manufacturer's instructions.

### *In vivo* Ubiquitination Assay

*In vivo* ubiquitination assays were performed according to a previously described protocol (7). Briefly, DLD-1 or RKO cells in six-well plates were transfected with 1 μg pcDNA-Ub-HA of ubiquitin-HA fusion protein (a gift from Dr. Xinjin Lin, Fujian Medical University, Fujian, China), 1 μg pcDNA-c-Myc with Flag tag at the C-terminal (Genechem, Shanghai, China), together with 40 pmol MEG3 siRNA or 1 μg pcDNA-*MEG3*. Twenty-four hours after transfection, the cells were treated with 30 μM MG132 (Sigma Aldrich) for 6 h and then lysed with RIPA lysis buffer. The cell lysates were then incubated with anti-HA magnetic beads (Bimake, Houston, TX, USA) overnight at 4°C. After washing, the proteins were eluted with SDS sample buffer. Eluted proteins were analyzed by Western blotting with anti-Flag (Sigma) antibody.

### Statistical Analysis

All data are presented as mean ± SEM. Student's *t*-test was used to compare data between two groups. Overall survival curves were plotted using the Kaplan–Meier method and analyzed with the log-rank test. Correlations were analyzed with Pearson r analysis. The clinicopathological characteristics of the 80 patients with CRC were subjected to logistic regression analysis. *P* < 0.05 was considered to denote statistical significance.

## Results

### *MEG3* Is Downregulated in CRC and Associated With Tumor Prognosis

**Figure 1:**
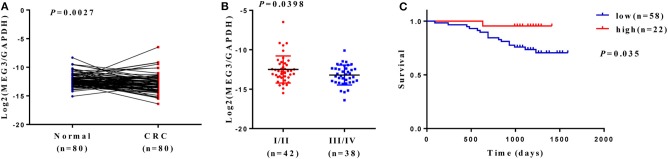
*Maternally expressed gene 3* (*MEG3*) is downregulated in colorectal cancer (CRC) tissues, and low expression of *MEG3* is associated with poor prognosis of patients with CRC. **(A)** Expression of *MEG3* is significantly lower in CRC tissues than in corresponding non-tumor tissues (*n* = 80). *MEG3* expression was quantitated by qRT-PCR and plotted with log2-MEG3/glyceraldehyde 3-phosphate dehydrogenase (GAPDH). **(B)** *MEG3* expression is negatively correlated with advanced CRC stage. **(C)** Kaplan–Meier survival curves showing an association between low expression of *MEG3* and short overall survival in patients with CRC.

Downregulation of *MEG3* has been observed in CRC tissues (8, 21). To further confirm this, we used RT-qPCR to measure *MEG3* expression in 80 CRC samples and the matched samples of adjacent normal mucosa. Consistent with others' findings, we found that there was significantly lower expression of MEG3 in CRC tissues (*P* < 0.05) than the corresponding adjacent normal mucosa (Figure 1A). Low expression of *MEG3* was significantly associated with advanced CRC clinical stage (*P* < 0.05) (Figure 1B). However, in our study, there was no significant association between the level of *MEG3* expression and various other clinical variables such as sex, age, and tumor size (Table 1). We also examined the relationship between *MEG3* expression and clinical outcomes, including overall survival. We divided the patients into two groups on the basis of the fold difference in expression of *MEG3* in the tumor and the corresponding adjacent normal tissue: *MEG3*-high group (fold difference >1, *n* = 22) and *MEG3*-low group (fold difference <1, *n* = 58) and then performed a Kaplan–Meier survival analysis and log-rank tests. We found that overall survival was significantly poorer in the *MEG3*-low group than that in the *MEG3*-high group (*P* < 0.05) (Figure 1C). These results indicate that *MEG3* expression is associated with cancer progression and poorer prognosis in patients with CRC.

### *MEG3* Inhibits Cell Proliferation and Invasion

**Figure 2:**
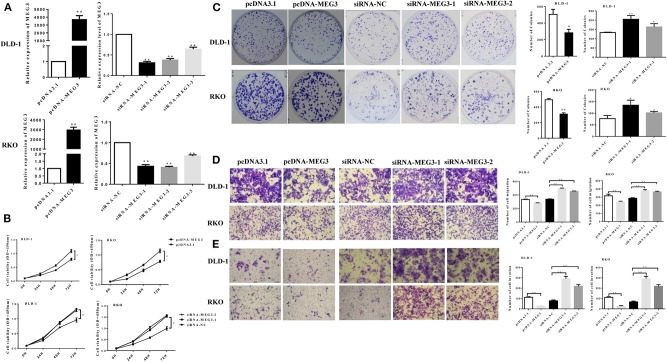
*Maternally expressed gene 3* (*MEG3*) inhibits colorectal cancer (CRC) cell proliferation and invasion. **(A)** *MEG3* expression as quantified by RT-qPCR in DLD-1 and RKO cells transfected with the indicated siRNAs or pcDNA-MEG3. **(B)** CCK-8 assays and **(C)** colony-forming growth assays were performed in DLD-1 and RKO cells transfected with *MEG3* siRNAs or pcDNA-MEG3. Transwell assays were performed to detect the migration **(D)** and invasion **(E)** of *MEG3* knockdown and overexpressing cells 36–48 h after transfection. Data are expressed as mean ± standard deviation from three independent experiments. *P < 0.05, **P < 0.01.

We next investigated the effect of MEG3 in CRC cells. We first transfected pcDNA-MEG3 vector or specific *MEG3* siRNAs into DLD-1 and RKO cells. As shown as Figure 2A, pcDNA-MEG3 transfection increased the expression of *MEG3*, whereas siRNAs specifically targeting *MEG3* decreased its expression. We next investigated the effect of proliferation of *MEG3* on CRC cell lines. Data from CCK-8 showed that *MEG3* overexpression reduced the viability of DLD-1 and RKO cells. In contrast, knockdown of *MEG3* significantly increased cell viability (Figure 2B). Colony formation assays revealed that an increase in *MEG3* significantly inhibited cell proliferation in both DLD-1 and RKO cell lines (282 ± 44 for DLD-1/pcDNA-MEG3 vs. 506 ± 61 for DLD-1/pcDNA, *P* = 0.0412; 308 ± 17 for RKO/pcDNA-MEG3 vs. 494 ± 16 for RKO/pcDNA, *P* = 0.0014), whereas *MEG3*-decreased cells had the opposite effect (205 ± 12 for DLD-1/siRNA-MEG3-1 vs. 133 ± 1 for DLD-1/siRNA-NC, *P* = 0.0036 and 164 ± 10 for DLD-1/siRNA-MEG3-2 vs. 133 ± 1 for DLD-1/siRNA-NC, *P* = 0.0388; 134 ± 13 for RKO/siRNA-MEG3-1 vs. 77 ± 8 for RKO/siRNA-NC, *P* = 0.0171 and 101 ± 3 for RKO/siRNA-MEG3-2 vs. 77 ± 8 for RKO/siRNA-NC, *P* = 0.0402) (Figure 2C).

The effects of *MEG3* on migration and invasion were then investigated. The results of the Transwell assays showed that overexpression of *MEG*3 significantly impaired CRC cell migratory and invasive ability, whereas knockdown of *MEG3* strengthened these abilities compared with control cells (Figures 2D,E).

Taken together, the above data suggest that *MEG3* inhibits CRC cell proliferation and invasion.

### *MEG3* Influences Glucose Metabolism in Colorectal Cancer Cells

**Figure 3:**
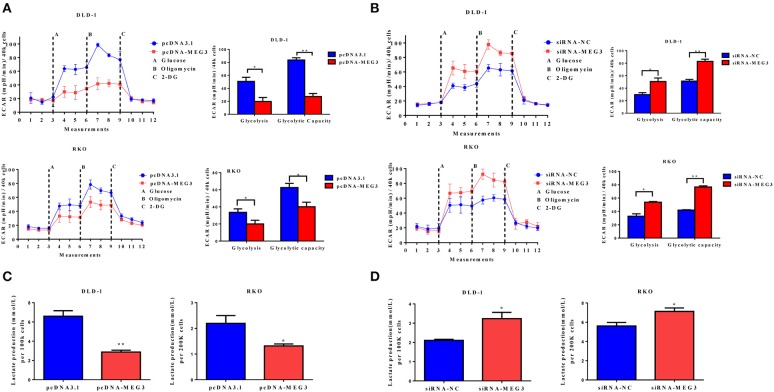
*Maternally expressed gene 3* (*MEG3*) influences glucose metabolism in colorectal cancer (CRC) cells. The extracellular acidification rate (ECAR) was measured by glycolysis stress tests in CRC cell lines with *MEG3* overexpression **(A)** and in those with *MEG3* knockdown **(B)**. Lactate production was measured in the culture medium of CRC cell lines with strong expression of *MEG3* **(C)** and weak expression of *MEG3* **(D)**. Data are expressed as mean ± standard deviation from three independent experiments. *P < 0.05, **P < 0.01.

Proliferation of tumor cells is reportedly usually accompanied by metabolic changes (11). Glycolysis is a hallmark of cancer cells. To investigate the role of MEG3 in glycolysis in CRC cells, we performed glycolysis stress test assays by using an XF analyzer to measure the ECAR. We found that *MEG3* overexpression significantly inhibited glycolysis and glycolytic capacity in DLD-1 and RKO cells (Figure 3A), whereas *MEG3* knockdown produced the opposite effect (Figure 3B). Moreover, we assessed cellular lactate production and found that *MEG3* overexpression inhibited lactate production (Figure 3C), whereas knockdown of *MEG3* by siRNA boosted lactate generation (Figure 3D). These data indicate that *MEG3* inhibits glucose metabolism in CRC cells.

### *MEG3* Reduces c-Myc Protein Stability

**Figure 4:**
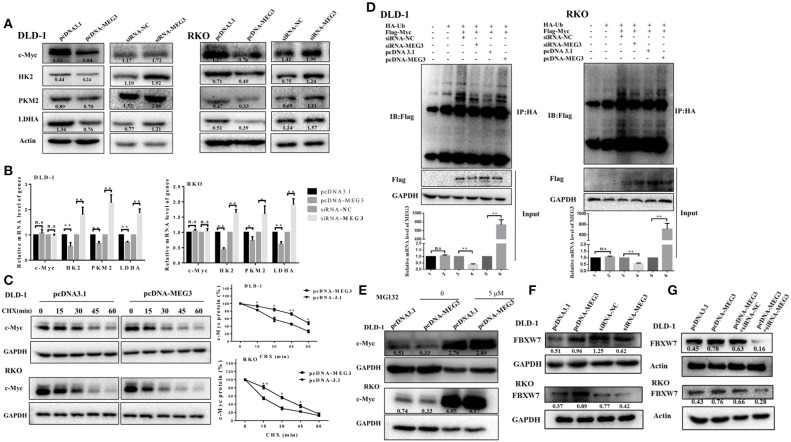
*Maternally expressed gene 3* (*MEG3*) promotes c-Myc protein degradation. Expression levels of c-Myc, HK2, PKM2, and LDHA were detected by Western blot **(A)** and qRT-PCR **(B)** analysis in *MEG3* overexpression and knockdown colorectal cancer (CRC) cell lines. **(C)** *MEG3* overexpression CRC cell lines were treated with 100 μg/ml of cycloheximide (CHX) and harvested at the indicated time points. c-Myc protein was detected by Western blot analysis, quantified by densitometry, and plotted against time to determine c-Myc stability. **(D)** CRC cells were transfected with pcDNA-c-Myc in combination with pcDNA-MEG3 in the presence of the HA-ubiquitin plasmid as indicated at the top. The cells were treated with MG132 (30 μM) for 6 h before harvesting, and the cell lysates were subjected to immunoprecipitation using anti-HA antibody. Ubiquitinated proteins were detected by Western blot with the anti-Flag antibody. **(E)** CRC cell lines that strongly express *MEG3* were treated with 5 μM of MG132 for 12 h, and c-Myc protein was detected by Western blot. **(F)** Expression of FBXW7 was detected by Western blot in CRC cells that strongly and weakly expressed *MEG3*. **(G)** Level of FBXW7 was measured by Western blot in the pcDNA-MEG3 cells with *MEG3* knockdown. Data are expressed as mean ± standard deviation from three independent experiments. *P < 0.05, **P < 0.01.

Previous studies have demonstrated that c-Myc is primarily responsible for metabolic reprogramming of cancer cells (13). We therefore next investigated whether c-Myc participates in *MEG3*-mediated inhibition of glycolysis. As shown in Figure 4A, we found that *MEG3* overexpression inhibited amounts of c-Myc protein and its targeted genes (*HK2, PKM2, LDHA*), whereas *MEG* siRNA did the opposite. The results of RT-qPCR indicated that amounts of mRNA of *HK2, PKM2*, and *LDHA* decreased in *MEG3*-overexpressed cells, whereas c-Myc did not change (Figure 4B), suggesting that *MEG3* decreases c-Myc expression in the protein level. To verify this, we performed CHX treatment and found that the half-life of c-Myc in pcDNA3.1 is longer than that in pcDNA-MEG3 (59.19 ± 3.39 min for DLD-1/pcDNA3.1 vs. 37 ± 4.2 min for DLD-1/pcDNA-MEG3, *P* = 0.0021; 31.34 ± 1.34 min for RKO/pcDNA3.1 vs. 17.15 ± 2.58 min for RKO/ pcDNA-MEG3, *P* = 0.001) (Figure 4C). These data showed that MEG3 overexpression reduced the c-Myc stability. To determine whether *MEG3* affects proteasomal degradation of c-Myc, we performed *In vivo* ubiquitination by transfecting pcDNA-Ub-HA and pcDNA-c-Myc together with pcDNA-MEG3 into DLD-1 or RKO cells in which we immunoprecipitated ubiquitin with anti-HA antibody and detected it by anti-Flag. We found that overexpression of *MEG3* increased polyubiquitination of c-Myc (Figure 4D). Treatment with MG132, a proteasome inhibitor to inhibit protein degradation through proteasome-dependent pathway, rescued c-Myc protein expression (Figure 4E), suggesting that *MEG3* affects c-Myc stability in proteasome-dependent degradation. Numerous studies have shown that E3 ubiquitin ligase of FBXW7 controls proteasome-mediated degradation of c-Myc (22). We therefore measured the expression of FBXW7 in *MEG3* overexpression and *MEG3*-knockdown cells and found that *MEG3* induced the expression of FBXW7 (Figure 4F). Meanwhile, knockdown of *MEG3* reversed the FBXW7 level induced by MEG3 overexpression in CRC cells (Figure 4G). These results indicate that *MEG3* may contribute to inhibiting glycolysis through promoting c-Myc degradation by increasing the amounts of FBXW7.

### *MEG3* Activated by Vitamin D Through Vitamin D Receptor

**Figure 5:**
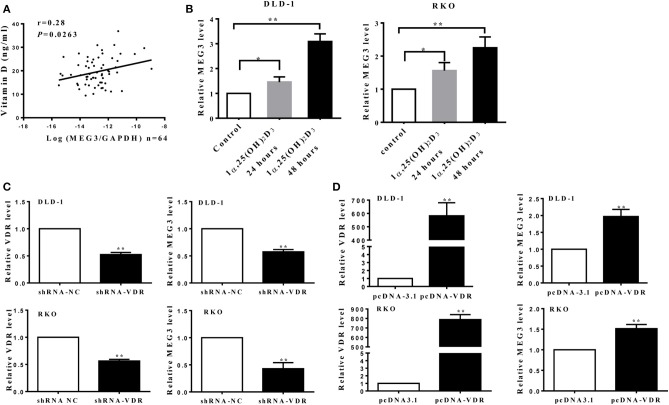
*Maternally expressed gene 3* (*MEG3*) is activated by vitamin D through vitamin D receptor (VDR). **(A)** Levels of MEG3 expression in colorectal cancer (CRC) tissues and serum concentrations of vitamin D were examined in 64 CRC patients, and the correlation between MEG3 and vitamin D was analyzed. **(B)** *MEG3* expression was measured with qRT-PCR after treatment with active vitamin D of 1α,25(OH)_2_D_3_ (20 nM) at the indicated times. *MEG3* expression was detected by qRT-PCR in CRC cells transfected with shRNA-VDR **(C)** or pcDNA-VDR **(D)**. Data are expressed as mean ± standard deviation from three independent experiments. *P < 0.05, **P < 0.01.

A previous study has shown that vitamin D can promote *MEG* transcription through binding VDR (10). To further investigate this, we first measured serum concentrations of vitamin D [25(OH)_2_D_3_] in samples from CRC patients and found that these concentrations correlate positively with *MEG3* expression in CRC tissues (Figure 5A). To further determine the effect of vitamin D on *MEG3* expression, qRT-PCR analysis was performed and showed that amounts of *MEG3* increased with vitamin D treatment and VDR overexpression but decreased with VDR knockdown (Figures 5B–D). Above all, our clinical and cellular data suggest that *MEG3* expression is indeed positively regulated by vitamin D through VDR.

### Vitamin D Receptor Knockdown Abolishes the Effect of *MEG3* on Glucose Metabolism

**Figure 6:**
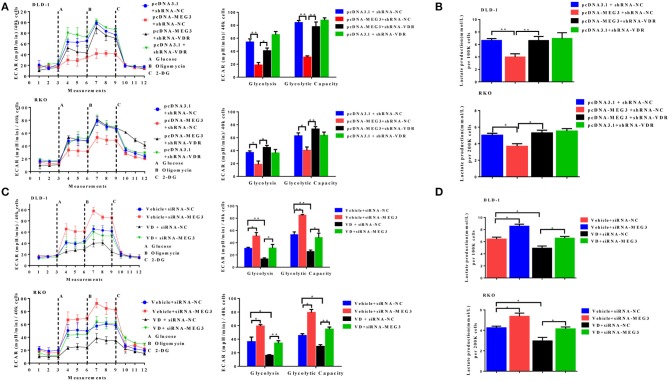
Vitamin D receptor (VDR) knockdown abolishes the effect of *maternally expressed gene 3* (*MEG3*) on glucose metabolism. The extracellular acidification rate (ECAR) (**A**, left panel) and lactate production **(B)** were measured in colorectal cancer (CRC) cell lines with *MEG3* overexpression and weak VDR expression (pcDNA-MEG3 + shRNA-VDR) or with *MEG3* overexpression and base VDR expression (pcDNA-MEG3 + shRNA-NC). CRC cells with low expression of MEG3 were treated with 1α,25(OH)_2_D_3_ (vitamin D) (VD + siRNA-MEG3) or vehicle (Vehicle + siRNA-MEG3), and the ECAR (**C**, left panel) and lactate production **(D)** were measured. Glycolysis and glycolytic capacity were analyzed using XF Glycolysis Stress Test software (right panels of **A,C**). Data are expressed as mean ± standard deviation from three independent experiments. *P < 0.05, **P < 0.01.

To further determine whether vitamin D or VDR affects the effects of *MEG3* on glucose metabolism, we performed glycolysis stress assays and lactate production assays. As shown in Figure 6A, both in DLD-1 and RKO cells, VDR knockdown greatly reversed *MEG3* overexpression-induced inhibition of glycolysis and glycolytic capacity. Lactate production also increased consistently after VDR knockdown in *MEG3*-overexpressed cells (Figure 6B). Previous studies have shown that vitamin D decreases glycolysis in various cancers, including CRC (23–25). We speculated whether MEG3 mediated vitamin D-induced inhibition of glycolysis. To investigate this possibility, we treated *MEG3*-knockdown cells with vitamin D and performed glycolysis stress assays and lactate production assays. The results showed that vitamin D treatment inhibited significantly the glycolysis and glycolytic capacity and decreased the lactate production of CRC cells (Figures 6C,D). These inhibitions are partially rescued by *MEG3* knockdown (Figures 6C,D). These data implicate that vitamin D inhibits glycolysis in CRC cells partially through the VDR/MEG3 pathway.

## Discussion

CRC had the third highest annual age-standardized incidence (2009–2013) worldwide, being 40.7 per 100,000 people with a mortality rate (2010–2014) of 14.8 per 100,000 persons (1, 2). However, the molecular mechanisms that underlie CRC tumorigenesis are yet to be fully elucidated. There is increasing evidence that lncRNAs acting as oncogenes or anti-oncogene factors have functions in tumorigenesis and tumor metastasis in CRC cells (5, 8, 26, 27).

*MEG3* is aberrantly expressed in multiple types of cancers and is assumed as a tumor suppressor (10, 28, 29). *MEG3* participates in carcinogenesis and cancer progression by regulating gene expression through chromatin modification, transcription, and posttranscriptional procession. For example, *MEG3*, as a competing endogenous RNA, reduces the invasiveness of human bladder cancer cells by competing with PHLPP2 mRNA for miR-27a (30); *MEG3* inhibits the proliferation of epithelial ovarian cancer cells by regulating ATG3 activity and inducing autophagy (31); and *MEG3* inhibits the proliferation and invasion of gallbladder cancer by associating with EZH2 and promoting its ubiquitination (32). Some recent studies have reported that the amounts of *MEG3* are significantly reduced in tissue and serum from patients with CRC and that this can serve as a prognostic marker in such patients (8, 28, 33). However, the role of *MEG3* in CRC remains largely unknown. In the present study, we also found that *MEG3* expression was lower in tumor tissue than adjacent normal tissues from patients with CRC (Figure 1A). We identified downregulation of *MEG3* in advanced stages of CRC, and this was closely associated with poor overall survival of the patients (Figures 1B,C). Functional studies demonstrated that *MEG3* inhibited CRC cell proliferation, migration, and invasion (Figure 2), which is consistent with previous studies. Moreover, we found that *MEG3* inhibited glycolysis in CRC cells (Figure 3).

Increased glycolysis is the main source of energy in cancer cells, which use this metabolic pathway to generate ATP (34), this being known as the Warburg effect because it was first described by Otto Warburg in the 1920s (35). Many studies have found that lncRNAs can regulate the Warburg effect to influence the growth and survival of cancer cells (36–38). The mechanism of lncRNA-mediated regulation is complex; however, many studies have found that lncRNAs can affect genes involved in glucose metabolism. It has been extensively documented that the oncogene of c-Myc is a key regulator of the Warburg effect by directly activating several glycolytic genes, including *LDHA, PKM2*, and others (39–41). Several studies have shown that lncRNAs regulate processes of glycolysis in cancer by altering amounts of c-Myc and, more specifically, by modifying the transcriptional patterns on c-Myc target genes. For example, lncRNA GLCC1 promotes glucose metabolism in CRC cells by protecting c-Myc from ubiquitination *via* direct interaction with heat shock protein (HSP)90 chaperone (42). LINC01123 promotes non-small-cell lung carcinoma (NSCLC) cell proliferation and aerobic glycolysis by increasing c-Myc mRNA expression with sponging miR-199a-5p (43). In the current study, we found that *MEG3* inhibited c-Myc expression and its target glycolytic genes including *HK2, PKM2*, and *LDHA* (Figures 4A,B). Expression of c-Myc is largely posttranslationally regulated by E3 ubiquitin ligase, which binds c-Myc to promote its degradation. In the current study, we found that *MEG3* inhibited the expression of c-Myc by promoting its ubiquitination, further increasing its degradation (Figures 4C–E). Moreover, the increase in ubiquitination of c-Myc that was induced by *MEG3* may result from upregulation of FBXW7 (Figure 4F). However, how *MEG3* regulates FBXW7 is not clear and requires further study.

Multiple studies have consistently shown an inverse association between serum vitamin D concentrations and risk of CRC (44–46). Our previous study also showed that vitamin D-deficient mice develop colonic inflammation (47). In the current study, we found a positive correlation between serum vitamin D concentration and *MEG3* expression in patients with CRC (Figure 5A); to the best of our knowledge, we are the first to document a correlation between *MEG3* and serum vitamin D. In addition, we showed that *MEG3* can be induced by vitamin D *via* VDR transcription factor (Figures 5B–D), which is consistent with previous research (10). Moreover, VDR knockdown and MEG3 knockdown retard inhibition of glycolysis induced by *MEG3* or vitamin D, respectively (Figures 6A–D). Interestingly, we found that glycolysis and glycolytic capacity and lactate production were consistently lower in VD + siRNA-MEG3 compared to vehicle + siRNA-MEG3 cells. Considering that vitamin D could regulate many genes by binding VDR, it might be possible that additional inhibitory mechanisms are involved in the glycolysis inhibited by vitamin D. Our data suggest that *MEG3* activated by vitamin D is partly responsible for the anti-CRC effect of vitamin D.

In conclusion, in this study, we found that *MEG3* is significantly decreased in CRC tissues and is positively associated with serum vitamin D concentrations in patients with CRC, indicating that it is a potential prognostic biomarker and therapeutic target for CRC. Vitamin D-activated *MEG3* suppresses aerobic glycolysis of CRC cells *via* degrading c-Myc, suggesting that vitamin D may have therapeutic value in the treatment of CRC.

Thus, the present data indicate that the anticancer function of vitamin D may be executed *via* the VDR/MEG3 pathway.

## Data Availability Statement

The raw data supporting the conclusions of this article will be made available by the authors, without undue reservation, to any qualified researcher.

## Ethics Statement

The studies involving human participants were reviewed and approved by the Research Ethics Committee of Nanjing Medical University [Approval ID: (2016)640]. The patients/participants provided their written informed consent to participate in this study.

## Author Contributions

XY designed the study and wrote the manuscript. SZ and LW developed the methodology and performed the analyses. YW collected the data.

### Conflict of Interest

The authors declare that the research was conducted in the absence of any commercial or financial relationships that could be construed as a potential conflict of interest.
